# Mindfulness exercise and mental health among university students: the sequential mediating roles of sleep regularity and self-control

**DOI:** 10.3389/fpsyg.2026.1783570

**Published:** 2026-03-27

**Authors:** Jifeng Dong, Qiuxian Ye, Taiping Li, Yafei Yuan, Yanqing Yan

**Affiliations:** 1 Guangdong University of Science and Technology, Dongguan, Guangdong, China; 2 Zhuhai College of Science and Technology, Zhuhai, Guangdong, China; 3 Army Special Operations College, Guilin, Guangxi, China

**Keywords:** mental health, mindfulness exercise, self-control, sleep regularity, university students

## Abstract

**Background:**

The mental health problem of college students has become an increasingly prominent public health problem. Mindfulness exercise (ME) is considered to be a promising way to improve psychological well-being. However, the underlying mechanisms by which mindfulness exercise affects mental health (MH) remain poorly understood. Based on the theory of self-regulation, this research explores the sequential mediating role of sleep regularity (SR) and self-control (SC) in the relationship between mindfulness exercise and mental health of college students.

**Methods:**

A cross-sectional survey was conducted among 470 Chinese undergraduate students using validated self-report instruments measuring mindfulness exercise, sleep regularity, self-control, and mental health. Data were analyzed using partial least squares structural equation modeling (PLS-SEM) with SmartPLS 4.1. Measurement reliability and validity were assessed, and bootstrapping procedures (5,000 resamples) were employed to test direct, indirect, and sequential mediation effects.

**Results:**

The measurement model demonstrated satisfactory reliability, convergent validity, and discriminant validity. Structural model results indicated that mindfulness exercise was positively associated with sleep regularity (*β* = 0.457, *p* < 0.001), sleep regularity was positively associated with self-control (*β* = 0.288, *p* < 0.001), and self-control was positively associated with mental health (*β* = 0.296, *p* < 0.001). Mediation analyses revealed that sleep regularity significantly mediated the relationship between mindfulness exercise and self-control, while self-control mediated the association between mindfulness exercise and mental health. Moreover, a significant sequential mediating effect of sleep regularity and self-control was observed in the relationship between mindfulness exercise and mental health.

**Conclusion:**

Mindfulness exercise was positively associated with MH among university students, and this association was linked to sleep regularity and self-control. The results enhance the theoretical understanding of the behavioral and self-regulation mechanisms linking mindfulness exercise and mental health, and provide practical implications for the development of integrated mental health promotion interventions targeting sleep behavior and self-regulation in higher education environments.

## 1 Introduction

MH has become a public health problem increasingly concerned by college students all over the world (Bantjes et al., 2022; Hernández-Torrano et al., 2020; Karyotaki et al., 2020). The transitional characteristics of adulthood, coupled with growing academic needs, social pressure and irregular lifestyles, make students face higher risks of psychological distress and impaired well-being (Gill, 2021; Granieri et al., 2021; Talley, 2024). Recent evidence suggests that suboptimal mental health during college not only impairs academic performance and interpersonal function, but also increases long-term psychological problems (Limone and Toto, 2022; Otieno, 2025; Zhang et al., 2024). Therefore, it has become the focus of MH research in the context of higher education to identify modifiable behavioral factors and clarify their internal mechanism.

Mindfulness practice, as a feasible and simple method to improve MH, has attracted more and more attention (Chen et al., 2023; Deshpande et al., 2024; González-Martín et al., 2023). Conceptually, mindfulness practice emphasizes immediate awareness, attention control and non judgmental acceptance in physical activities, thereby integrating cognitive and behavioral self-regulation processes (Schuman-Olivier et al., 2020). Empirical research shows that mindfulness practice is related to lower levels of stress, anxiety and depression symptoms, as well as enhanced emotional regulation and subjective well-being (Ma and Xiang, 2023; Xiong et al., 2025). Despite these promising findings, existing studies mainly focus on the direct relationship between mindfulness related practice and mental health outcomes, while the exploration of potential behavior and self-regulation mechanisms is not enough, especially in non clinical students.

From the perspective of self-regulation, sleep behavior is a key but often neglected way to link mindfulness training with mental health. Sleep regularity refers to the consistency of sleep wake patterns throughout the day, maintaining the stability of circadian rhythm and psychological function (Hartstein et al., 2025; Li et al., 2025). Irregular sleep patterns are common among college students, which are related to impaired emotion regulation, decreased cognitive control ability and increased psychological pain. Mindfulness exercise may enhance body awareness, reduce physiological arousal, and cultivate adaptive daily behavior habits (Ding et al., 2020; Pickett et al., 2024) to improve sleep patterns. However, the empirical evidence for directly testing sleep regularity as a mediating mechanism between mindfulness exercise and mental health is still limited.

In addition to sleep-related processes, self-control is the core psychological resource for adaptive function and mental health. SC refers to the ability to regulate impulses, maintain attention, and align behavior with long-term goals (Birech and Onyango, 2025; Şimşir Gökalp, 2023). Higher self-control level, better emotional stability, healthier lifestyle and lower susceptibility to mental health problems (He et al., 2023; Şimşir Gökalp, 2023). Theoretical and empirical studies have shown that regular and structured sleep patterns help to restore self-regulation resources, thereby enhancing self-control (Liu et al., 2025). However, few studies have integrated sleep patterns and self-control systems into a unified framework to explain how mindfulness exercise translates into improved mental health.

In response to these gaps, this study proposes a sequential mediation model, that is, mindfulness exercise may be indirectly associated with mental health through sleep regularity and self-control. Based on the theory of self-regulation, the model believes that mindfulness exercise promotes more regular sleep behavior, which may in turn be related to higher self-control and ultimately promotes better mental health outcomes. This paper uses partial least squares structural equation model to empirically test the sequential path of college students, trying to expand the existing literature from several aspects. First, clarify the behavior and psychological mechanism between mindfulness exercise and mental health, and promote theoretical understanding. Secondly, it emphasizes that sleep patterns, as a key behavioral channel, have a downstream effect on self-regulation resources through mindfulness practice. Third, provide practical enlightenment for the development of comprehensive mental health promotion strategies of mindfulness practice, sleep behavior and self-control for college students.

## 2 Literature review and research hypotheses

In this study, the concept of mindfulness practice is to apply mindfulness related physical activities in the process of attention and self-regulation. Although the exercise mindfulness scale (Thienot et al., 2014) was originally developed in the context of exercise, its core dimensions, such as Refocus, current consciousness and nonresponsiveness to internal distractions, reflect the generalized self-regulation ability, rather than exercise specific performance skills. These processes are theoretically consistent with the broader definition of mindfulness, which is context dependent self-regulation in goal-directed activities (Schuman-Olivier et al., 2020). Therefore, the scale is used to record the mindfulness related regulatory participation in the process of College Students’ participation in sports, rather than the performance of competitive sports itself (González-Martín et al., 2023; Johnson et al., 2023).

Self regulation theory provides an overall framework for understanding how individuals monitor, evaluate and adjust their behavior, cognition and emotion to achieve expected goals (Carver and Scheier, 2001; Stojanovic and Wood, 2024). The theory assumes that self-regulation operates at interrelated levels, including behavioral regulation (such as daily life), physiological regulation (such as day and night stability), and psychological regulation (such as impulse control and executive function) (McClung, 2011; Nigg, 2017). From the perspective of resource base, the regulatory ability is not static, but depends on the stability of daily behavior patterns and the recovery of physiological resources. Consistent routines improve regulatory efficiency, while non-standard behaviors may undermine executive control and emotional stability (Perez et al., 2022). Within this framework, mindfulness practice can be conceptualized as an upstream regulatory input to enhance attention monitoring and behavioral awareness. Sleep regularity represents self-regulation of behavior and reflects the stability of daily rhythm. Self control reflects high-order psychological regulation supported by stable physiological and behavioral basis. Therefore, the results of mental health can be understood as the downstream indicators of successful multi-level self-regulation.

Based on this view of regulatory cascade, this study proposes that mindfulness exercise affects mental health through continuous behavioral and psychological regulation mechanisms.

### 2.1 Mindfulness exercise and sleep regularity

From the perspective of self-regulation, behavioral consistency is the basic component of regulatory function. Mindfulness practice enhances awareness and attention monitoring between moments, thus promoting consistency between intention and daily behavior. This increased awareness can reduce cognitive and physiological hyper awakefulness, thereby promoting greater stability of the sleep wake process (Xie et al., 2023).

As a form of behavioral regulation, sleep regulation reflects the consistency of circadian rhythm and daily structure (Barrett et al., 2020). According to the theory of self-regulation, a stable behavior pattern preserves regulatory resources and prevents the exhaustion of executive functions. Therefore, mindfulness practice is expected to promote sleep regularity as an obvious manifestation of improving behavioral self-regulation.

*H1*: Mindfulness exercise is positively associated with sleep regularity.

### 2.2 Sleep regularity and self-control

Self control represents a person’s ability to regulate impulses, maintain goal-directed behavior, and align actions with long-term goals (Stojanovic and Wood, 2024). As the core resource of self-regulation, self-control is related to emotional stability, adaptive coping and mental health (Pan et al., 2023; Şimşir Gökalp, 2023). The theoretical model of self-regulation emphasizes that under physiological or cognitive stress conditions, self-control resources are limited and easy to be exhausted. Sleep regularity is essential for recuperation and self-regulation (Castiglione-Fontanellaz et al., 2023).

Within the regulatory framework, psychological regulation depends on physiological stability. The regular sleep wake cycle stabilizes the circadian rhythm and supports the neurocognitive process of executive control. The regular interruption of sleep can damage the attention control and inhibition function, thus weakening the ability of self-regulation (Wang W. et al., 2024).

Therefore, sleep regulation is the behavioral and physiological basis for establishing psychological self-control. In this sense, self-control is not independent, but supported by consistent behavioral regulation.

*H2*: Sleep regularity is positively associated with self-control.

### 2.3 Self-control and mental health

Mental health includes emotional health, cognitive function and effective social adaptation (Cohn-Schwartz, 2020). Under the framework of self-regulation theory, self-control is considered to be an important psychological resource for individuals to manage stress, regulate emotions and maintain adaptive behavior. Individuals with high self-control can better inhibit maladaptive impulses, re-evaluate stressors, and adhere to goal-oriented coping strategies, which all contribute to more favorable MH outcomes (Chen et al., 2025; He et al., 2023).

Empirical evidence consistently shows that higher self-control is associated with lower levels of psychological distress and higher levels of psychological well-being. In the university environment, self-control has been shown to buffer the negative impact of academic stress, reduce the vulnerability to maladaptive coping behaviors, and promote emotional stability (Xiang et al., 2024; Zhang et al., 2024). On the contrary, defects in self-control were associated with increased stress sensitivity, mood disorders, and poor mental health indicators. Therefore, self-control is expected to become a proximal psychological mechanism for the transformation of behavioral and lifestyle factors into mental health outcomes.

*H3*: Self-control is positively associated with mental health.

### 2.4 The mediating role of sleep regularity

Although mindfulness movement is associated with enhanced self-regulation (Ojell et al., 2023), the behavioral process of this process is still unclear. Sleep regulation is a reasonable regulation mechanism, because it reflects the transformation of internal self-consciousness to consistent daily behavior. By cultivating more attention control and body consciousness, mindfulness training can promote individuals to adopt a more stable sleep wake mode, thus supporting the recovery of self-regulated resources.

From this perspective, sleep patterns play a behavioral mediating role between mindfulness training and self-control. Mindfulness practice has no direct effect on self-control. It may first shape sleep related behaviors, thus creating good physiological and cognitive conditions for the effective operation of self-control.

*H4*: Sleep regularity mediates the relationship between mindfulness exercise and self-control.

### 2.5 The mediating role of self-control

In addition to behavioral pathways, mindfulness practice may also affect mental health by strengthening internal regulation. Mindfulness practice is theoretically considered to enhance attention regulation and reduce impulsive response, both of which are core components of SC. The improvement of SC ability in turn can promote more effective emotional regulation and adaptive coping, so as to promote better mental health.

Although mindfulness practice is usually associated with psychological benefits (Shankland et al., 2021; Strohmaier et al., 2021), its effect is unlikely to occur independently of the self-regulation mechanism. Therefore, self-control may be the psychological channel for mindfulness training to affect the results of mental health.

*H5*: Self-control mediates the relationship between mindfulness exercise and mental health.

### 2.6 Sequential mediation of sleep regularity and self-control

Drawing on Self-Regulation Theory, regulatory processes are hierarchically organized, such that behavioral regulation supports psychological regulation, which in turn influences emotional outcomes. Mindfulness exercise is expected to first enhance behavioral consistency (sleep regularity), which stabilizes physiological functioning and preserves regulatory resources. Stabilized regulatory resources then strengthen self-control capacity, enabling more effective emotion regulation and adaptive coping. Ultimately, enhanced self-control contributes to better mental health.

This continuous process reflects a cascade of regulation: attention regulation → behavior regulation → psychological regulation → mental health. Therefore, sleep regularity and self-control are not an independent mediator, but a continuous component of a multi-level regulatory mechanism.

*H6*: Sleep regularity and self-control sequentially mediate the relationship between mindfulness exercise and mental health.

**Figure 1:**
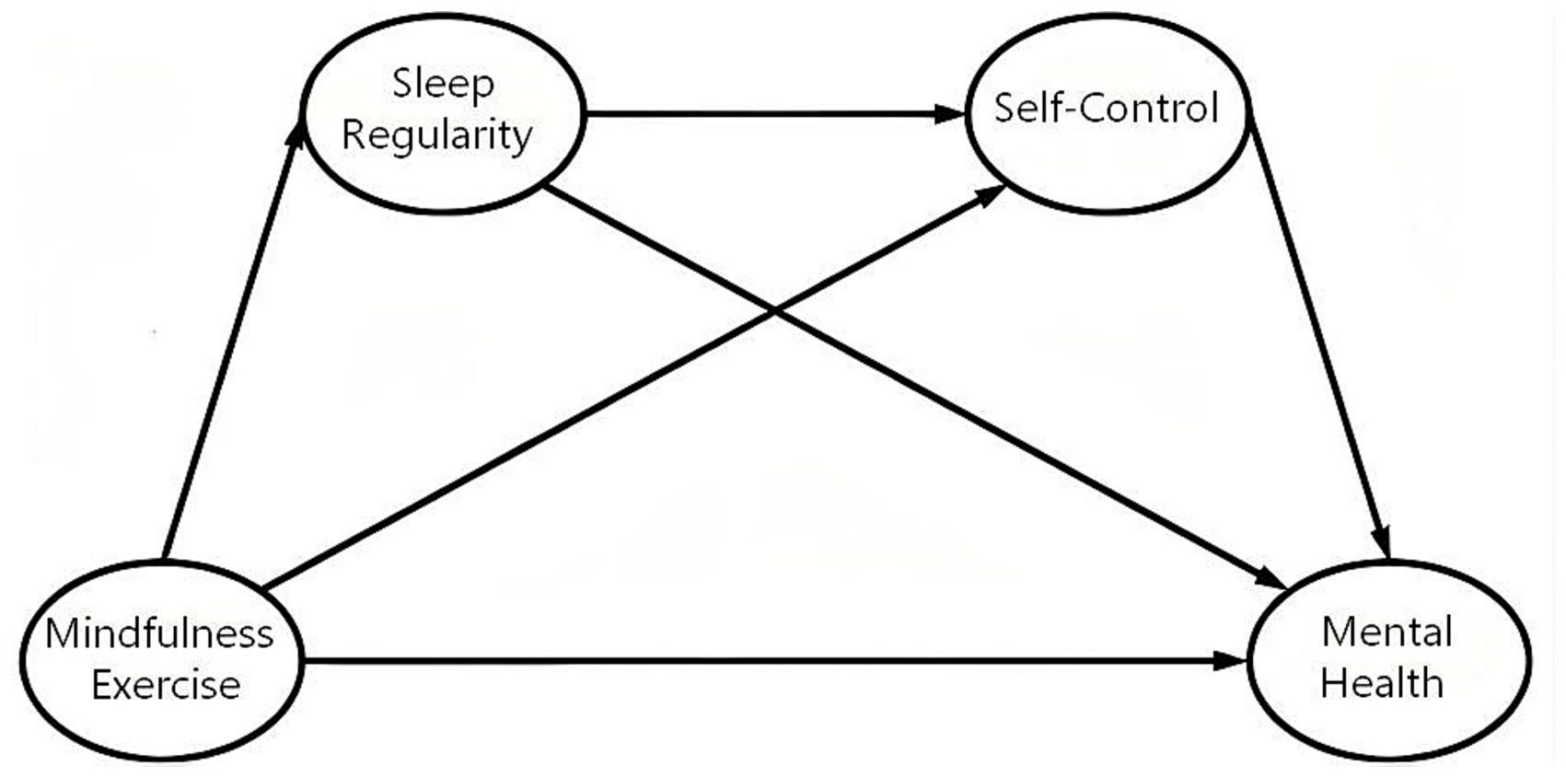
Hypothesized model.

The final conceptual model summarizes all the proposed direct and intermediary relationships, as shown in Figure 1.

## 3 Methods

### 3.1 Participants

This study was conducted from December 2025 to January 2026 to explore the relationship between mindfulness exercise, sleep regularity, self-control and mental health. Cluster random sampling was used in this study. The complete undergraduate class is regarded as a natural sampling group. First, a comprehensive list of all undergraduate courses offered in the current academic year was obtained from the Academic Affairs Office of the University. Classes that meet the following criteria are considered qualified: (1) full time undergraduate degree; (2) Status of the last year of non graduation to avoid absence related to internship; (3) Students did not participate in similar research. Secondly, the identification number is assigned to the qualified classes, and the classes of each academic year are selected proportionally using the random number generator to ensure representativeness. Then, contact the course instructors of the selected classes and invite them to help distribute the questionnaire.

In each selected class, all registered students are invited to volunteer. No additional screening was conducted at the teacher level, as teachers were recruited based on randomly selected classes rather than individuals.

A total of 520 questionnaires were distributed. A total of 498 questionnaires were collected, and the recovery rate was 95.77%. After data screening, 28 questionnaires were excluded due to invalid or repeated answers. Finally, 470 valid questionnaires were retained for analysis, and the effective rate was 90.38%.

Invalid answers are defined according to the following criteria: questionnaires with a large number of missing data (incomplete items more than 10%); Linear response mode (all items have the same answer); The completion time was significantly lower than the reasonable threshold (less than 2 min); Identify duplicate submissions through duplicate IP addresses or the same response pattern.

When duplicate submissions were detected, only the first valid entry was retained. All data screening procedures were completed prior to statistical analysis. Within the final sample, 213 participants were male (45.32%) and 257 were female (54.68%). Most participants were between 18 and 20 years old (73.83%). Third-year undergraduates constituted 44.26% (*n* = 208), sophomores 39.79% (*n* = 187), and freshmen 15.74% (*n* = 74).

Inclusion and Exclusion Criteria.

Participants were included if they met the following criteria:

1. Full time undergraduate enrollment;
2. Between 18 and 23 years of age;
3. Able to read and understand Chinese;
4. Voluntarily agree to participate.

Participants were excluded if they:

1. Is a senior student engaged in off campus internship;
2. A duplicate response identified by IP address or the same response pattern has been submitted.

#### 3.1.1 Sample size determination

Prior to data collection, prior power analysis was performed using G*Power 3.1 to determine the minimum sample size required to detect the size of moderate effects in a multiple regression model. Assuming that the effect size f^2^ = 0.15, the significance level *α* = 0.05, and the statistical efficacy is 0.80, the most complex endogenous structure has at most two predictors, and the minimum sample size calculated is 68 participants.

The final valid sample of 470 participants basically exceeded this minimum requirement, ensuring that there is sufficient statistical capacity to test the hypothetical effect.

When the scheduled survey period is over and the target number of questionnaires (*n* = 520) issued has been completed, the recruitment of participants will stop. There is no temporary analysis to influence the recruitment decision.

### 3.2 Measures

All the tools used in this study were previously published and validated self-report scales. There is no newly developed questionnaire in this study. The scales have been widely used in previous studies and have shown satisfactory psychometric properties. The following provides detailed entry sources and representative sample entries, as well as complete references. All the original instruments were developed in English. In order to ensure linguistic and conceptual equivalence, standardized translation and back translation procedures are followed according to the established norms of cross-cultural adaptation.

Firstly, two bilingual researchers independently translated the original English scale into Chinese. After discussion and coordination, a preliminary Chinese version was formed by comparing and synthesizing the two translated versions. Secondly, the merged Chinese version was re translated into English by an independent bilingual translator who had no knowledge of the original project. Compare the translated version with the original scale to ensure semantic consistency.

Subsequently, a panel of experts consisting of two psychologists and one sports science researcher reviewed the translation project to assess its clarity, cultural appropriateness, and conceptual equivalence. Minor adjustments were made to the wording when necessary to improve readability for college students.

Finally, a pilot test was conducted on 30 undergraduate students to evaluate project understanding and clarity of response. No significant ambiguity was found. The final Chinese version was subsequently used for formal data collection.

### 3.3 Mindfulness exercise

Mindfulness exercise was measured using a previously published and validated scale developed by Thienot et al. (2014). A typical example is, ‘When I became aware of my tension, I was able to quickly refocus my attention on what I should be focusing on.’ The scale has excellent reliability and validity. In this investigation, the *α* coefficient is 0.834.

Although the sports mindfulness scale was initially verified in athletes, subsequent studies showed that its factor structure reflected the process of general attention control and self-regulation, and was suitable for fields outside the elite sports background. In this study, confirmatory measurement analysis supported satisfactory reliability and construct validity (CR = 0.883; AVE = 0.603; HTMT value<0.85) in non athlete college samples, indicating that the scale played an appropriate role in this population. The wording of these items refers to attention and emotion regulation during physical activities, rather than competitive situations, which is consistent with the operational definition of mindfulness exercises used in this study.

### 3.4 Self-control

SC was measured using a previously published and validated scale developed by Manapat et al. (2021). A typical example is, “I can concentrate very well.” The scale has excellent reliability and validity. In this investigation, the α coefficient is 0.876.

### 3.5 Sleep regularity

Sleep regularity was measured using a previously published and validated scale developed by Yan et al. (2024). A typical example is “My nightly sleep duration is approximately the same.” The scale has good reliability and validity. In this study, the α coefficient is 0.818.

### 3.6 Mental health

Mental health was measured using a previously published and validated scale developed by Lukat et al. (2016). It is often used to assess the overall state of college students’ emotions, cognition, behavior, and social function (e.g., “I live a happy life; I am confident to express my opinions”). Cronbach’s alpha was 0.893. The scale showed good reliability.

### 3.7 Measurement format and scoring

All indicators are composed of closed items on Likert five point scale (1 = strongly disagree; 5 = strongly agree). The comprehensive score is calculated by averaging the reserved items of each structure. This scoring method retains the original scale measurement, which is convenient for interpretation in structural equation modeling. The higher the score, the higher the level of mindfulness exercise participation, sleep regularity, self-control and mental health. In PLS-SEM analysis, all variables were modeled as continuous reflection structures.

### 3.8 Item reduction and measurement specification

Although the initial mindfulness exercise scale (15 items) and short self-control scale (13 items) included more items, this study used a set of simplified indicators for structural modeling.

Specifically, in the evaluation stage of PLS-SEM measurement model, items with low factor load (<0.70), cross loadings or conceptual redundancies were removed according to the established guidelines for reflection measurement model (Hair et al., 2019). The retained items have good reliability and convergent validity.

The final measurement specification ensures that each potential structure is represented by at least three reflective indicators, meeting the minimum identification requirements for structural equation modeling (Bollen, 1989). Previous studies have shown that the retention of three to seven good indicators per structure is sufficient to capture the meaning of the structure meaning without compromising validity (Bollen and Lennox, 1991; Jarvis et al., 2003; Nunnally and Bernstein, 1994).

Importantly, item reduction did not alter the conceptual domain of the constructs, as retained items adequately represented the theoretical dimensions of mindfulness-related attentional regulation and self-control capacity.

### 3.9 Procedure

In this study, we used the mindfulness exercise, self-control, sleep regularity and mental health scales to collect data through online questionnaires. All subjects agreed to sign the informed consent form before starting to fill out the online questionnaire. After the participants completed all the items, the test data was automatically generated. The procedure was approved by the Ethics Committee of Guangdong University of Science and Technology.

### 3.10 Statistical analysis

This study uses SPSS 29.0 software for descriptive statistics on the data. Additionally, we will use SmartPLS 4.1 software for the measurement model and structural evaluation during the data analysis process. This work applies PLS-SEM. This survey chose this software for analysis because it has been successful in evaluating validity and reliability and confirming or rejecting hypotheses (Martínez-Falcó et al., 2024). In terms of analytical advantages, partial least squares can simultaneously estimate the path coefficients of the specified model and the loadings of individual items. Consequently, it enables researchers to circumvent biased and inconsistent parameter estimates (Loureiro et al., 2013) and is applied to more advanced and complex models (Becker et al., 2012).

## 4 Results

### 4.1 Descriptive statistics

Descriptive statistics and inter-construct correlations are presented in Table 1. The mean scores ranged from 3.35 to 3.71, indicating moderate to moderately high levels across all constructs. Standard deviations ranged between 0.58 and 0.73, suggesting sufficient variability for structural modeling. Skewness values ranged from −0.59 to −0.11 and kurtosis values ranged from 0.36 to 1.14, all within acceptable limits (|skewness| < 2; |kurtosis| < 7), indicating no serious deviations from normality. All inter-construct correlations were positive and moderate in magnitude (*r* = 0.33–0.55), providing preliminary support for the hypothesized relationships while suggesting no multicollinearity concerns.

**Table 1:** Descriptive Statistics and Correlations.

Variable	M	SD	Skewness	Kurtosis	1	2	3	4
1. ME	3.50	0.62	−0.31	0.36	1.000			
2. MH	3.71	0.58	−0.11	0.54	0.550	1.000		
3. SC	3.35	0.62	−0.35	1.14	0.439	0.484	1.000	
4. SR	3.40	0.73	−0.59	0.57	0.457	0.332	0.429	1.000

### 4.2 Common method Bias test

Given that all variables were collected using self-report questionnaires from the same respondents, common method bias (CMB) was assessed using both Harman’s single-factor test and the full collinearity assessment approach.

First, Harman’s single-factor test was conducted using SPSS 29.0 through unrotated principal component analysis including all measurement items. The results showed that four factors with eigenvalues greater than 1 were extracted. The first unrotated factor accounted for 29.84% of the total variance, which is below the critical threshold of 40%, suggesting that common method bias is unlikely to be a serious concern (Harman, 1976; Podsakoff et al., 2003).

Second, following Kock (2015), a full collinearity assessment was conducted in SmartPLS. The variance inflation factor (VIF) values ranged from 1.000 to 1.392, all below the conservative threshold of 3.3, further indicating that common method bias does not pose a significant threat in this study (Kock, 2015).

### 4.3 Measurement model

According to Table 2, all constructs show acceptable reliability and convergent validity. Specifically, the Cronbach’s *α* value was between 0.818 and 0.893, and the CR value was between 0.880 and 0.916, both exceeding the recommended threshold of 0.70. AVE values were between 0.603 and 0.646, higher than the minimum cutoff of 0.50, indicating satisfactory convergent validity. In addition, according to the standard, item factor loadings should exceed 0.7 (Hair, 2014). Consequently, the overall convergent validity remains satisfactory (Hair et al., 2019). The HTMT values in Table 3 ranged from 0.381 to 0.631, which are all below the conservative threshold of 0.85. This demonstrates that each construct is empirically distinct and meets the requirements of discriminant validity (Hair, 2014). As indicated in Table 4, the square roots of the AVEs (diagonal values) were all greater than the inter-construct correlations (off-diagonal values). These results further confirm the discriminant validity of the measurement model (Hair, 2014).

**Table 2:** Reliability and validity.

Constructs	Items	Outer loadings	Cronbach’α	CR	AVE
Mindfulness exercise	ME 1	0.812	0.834	0.883	0.603
	ME 2	0.673			
	ME 3	0.822			
	ME 4	0.760			
	ME 5	0.807			
Self-Control	SC 1	0.801	0.876	0.907	0.618
	SC 2	0.807			
	SC 3	0.744			
	SC 4	0.774			
	SC 5	0.766			
	SC 6	0.824			
Sleep regularity	SR 1	0.809	0.818	0.880	0.646
	SR 2	0.768			
	SR 3	0.840			
	SR 4	0.797			
Mental health	MH 1	0.827	0.893	0.916	0.610
	MH 2	0.714			
	MH 3	0.818			
	MH 4	0.759			
	MH 5	0.777			
	MH 6	0.791			
	MH 7	0.777			

**Table 3:** Discriminant validity (HTMT Criterion).

Variable	ME	MH	SC	SR
ME				
MH	0.631			
SC	0.508	0.541		
SR	0.548	0.381	0.497	

**Table 4:** Discriminant validity (Fornell-Larcker Criterion).

Variable	ME	MH	SC	SR
ME	**0.777**			
MH	0.550	**0.781**		
SC	0.439	0.484	**0.786**	
SR	0.457	0.332	0.429	**0.804**

Bold values on the diagonal represent the square root of the average variance extracted (AVE) for each construct.

### 4.4 Structural model

The VIF values were examined to assess potential collinearity issues among the constructs. As shown in Table 5, the VIF values for ME, MH, SC, and SR ranged between 1.000 and 1.392. All values were well below the commonly accepted threshold of 3.3, indicating that multicollinearity was not a concern in the structural model. This result confirms that the predictor variables were sufficiently independent and suitable for subsequent path analysis (Hair, 2014; Sun et al., 2024).

**Table 5:** Collinearity test.

Variable	ME	MH	SC	SR
ME		1.392	1.265	1.000
MH				
SC		1.349		
SR		1.376	1.265	

The PLS guidance technique with a sample size of 5,000 was used to evaluate the size and importance of the model path coefficient (Sun et al., 2024). Table 5 summarizes the test results. In the structural model, the significance test aims to determine the influence of exogenous variables on endogenous variables.

The explanatory power of the structural model was assessed using R^2^ and adjusted R^2^ values. As shown in Table 6, sleep regularity (SR) yielded an R^2^ of 0.209 (Adjusted R^2^ = 0.208), self-control (SC) demonstrated an R^2^ of 0.259 (Adjusted R^2^ = 0.255), and mental health (MH) showed an R^2^ of 0.376 (Adjusted R^2^ = 0.372). According to Hair et al. (2019), these values indicate small to moderate explanatory power (Hair et al., 2019).

**Table 6:** Explanatory Power.

Endogenous Variable	*R* ^2^	Adjusted *R*^2^
Sleep regularity (SR)	0.209	0.208
Self-control (SC)	0.259	0.255
Mental health (MH)	0.376	0.372

Model fit was further evaluated using SRMR, d_ULS, and d_G indices. The SRMR value was 0.061, which is below the recommended threshold of 0.08, indicating acceptable model fit. The d_ULS (0.932) and d_G (0.335) values were within acceptable limits. The normed fit index (NFI) was 0.829, exceeding the acceptable cutoff of 0.80, further supporting adequate model fit (see Table 7).

**Table 7:** Model Fit Indices.

Model fit index	Saturated model	Estimated model
SRMR	0.061	0.061
d_ULS	0.932	0.932
d_G	0.335	0.335
NFI	0.829	0.829

Overall, the results suggest that the structural model demonstrates satisfactory explanatory power and acceptable global model fit.

**Figure 2:**
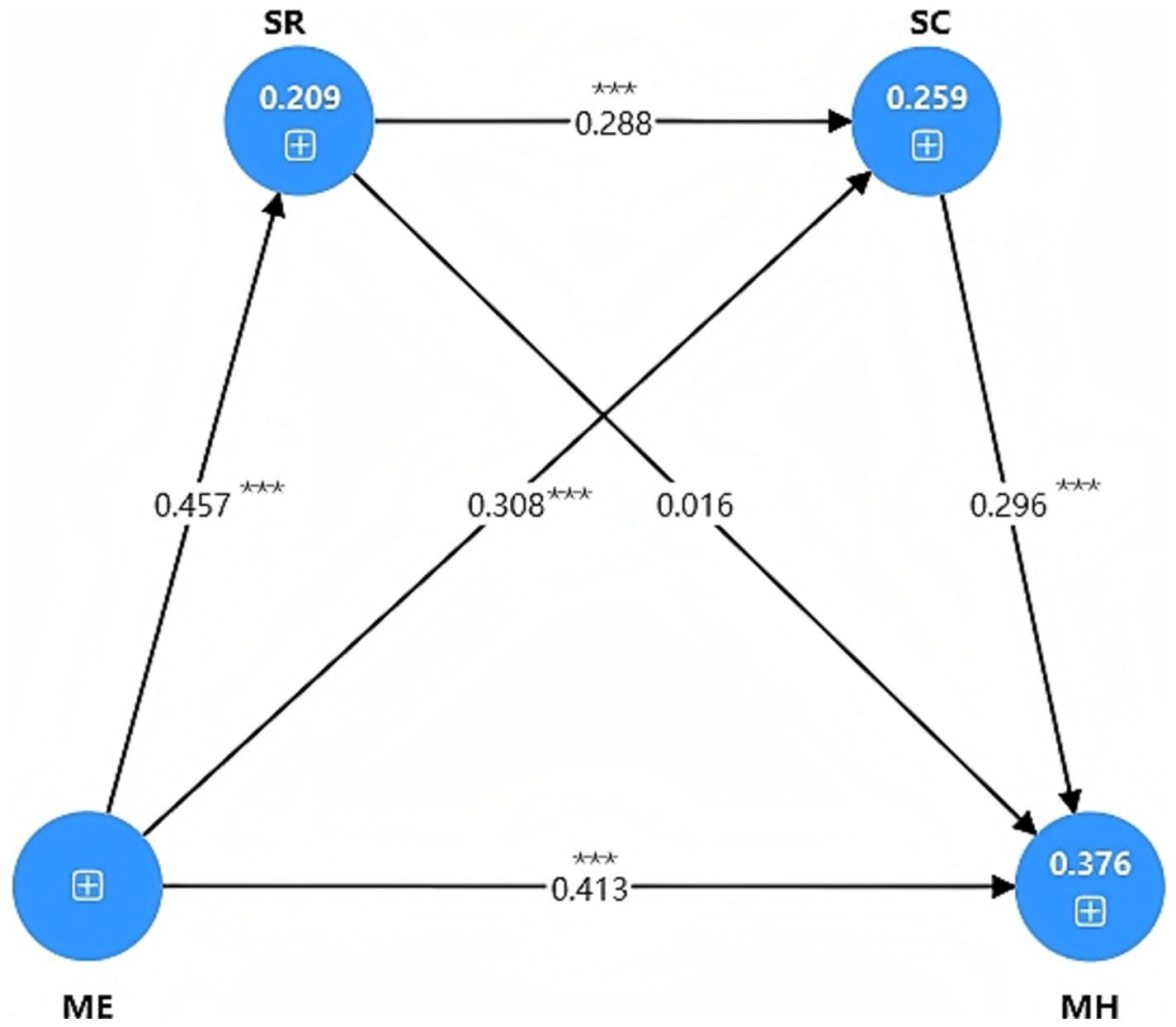
The SEM illustrates the relationships among ME, SR, SC, and MH. ***p < 0.001; **p < 0.01; *p < 0.05.

The structural model was evaluated to test the hypothetical relationship between ME, SR, SC and MH. As shown in Table 8 and Figure 2, the direct paths of all hypotheses are positive and statistically significant. Specifically, ME significantly predicted SR (*β* = 0.457, *p* < 0.001). Similarly, SR significantly predicted SC (*β* = 0.288, *p* < 0.001). Finally, SC significantly predicted MH (*β* = 0.296, *p* < 0.001).

**Table 8:** Path hypothesis testing.

Hypothesis	Original sample (β)	2.50%	97.50%	*t*	*p*	Results
ME → SR	0.457	0.373	0.540	10.737	0.000	Support
SR → SC	0.288	0.179	0.397	5.186	0.000	Support
SC → MH	0.296	0.202	0.393	5.952	0.000	Support

### 4.5 Mediation analysis

Bootstrap mediating effect was used to test the mediating effect between ME, SR, SC, and MH. The results are summarized in Table 9. First, mindfulness exercise had a significant indirect effect on self-control through sleep regularity (*β* = 0.132, *t* = 4.554, *p* < 0.001), indicating that sleep regularity had a significant mediating effect. Second, SC has a significant mediating effect in the relationship between mindfulness exercise and MH. The indirect effect of mindfulness exercise on mental health through SC was significant (*β* = 0.091, *t* = 3.994, *p* < 0.001). Finally, sequential mediation analysis revealed that mindfulness exercise through sleep regularity and self-control (ME → SR → SC → MH; *β* = 0.039, *t* = 3.556, *p* < 0.001) had a significant indirect effect on MH, which supported the existence of a significant sequential mediating path.

**Table 9:** Mediating analysis.

Relationship	Indirect effect	2.5%	97.5%	t	*p*	Direct effect	Type of mediation
ME → SR → SC	0.132	0.078	0.192	4.554	0.000	0.308	Partial mediation
ME → SC → MH	0.091	0.051	0.140	3.994	0.000	0.413	Partial mediation
ME → SR → SC → MH	0.039	0.021	0.063	3.556	0.000	0.413	Partial mediation

## 5 Discussion

Importantly, the proposed model was theory-driven rather than purely data-driven, as the hypothesized sequential pathway was derived from Self-Regulation Theory prior to empirical testing. Overall, the empirical results of this study consistently support the proposed hypothesis and provide robust evidence for the serial mediation model. However, given the cross-sectional design, these findings should be interpreted as association rather than causality, and future longitudinal studies need to establish a time-priority relationship between variables. First, mindfulness practice was found to be positively correlated with sleep patterns, supporting H1. This finding suggests that mindfulness practice is not only a physical activity, but also a self-regulation exercise, which may reflect stronger physical consciousness and attention regulation, which may be related to more stable daily sleep wake habits. Sleep regularity was positively correlated with self-control and H2 support. These results suggest that maintaining a stable sleep wake rhythm may play an important role in restoring and protecting self-regulation resources, thereby improving individual self-control ability. Thirdly, SC is significantly positively correlated with mental health, which supports the H3 hypothesis. This finding emphasizes that SC is an important psychological resource for college students, which contributes to the emotional stability, adaptive function and overall mental health of college students.

In terms of mediating effect, sleep regularity plays an important mediating role in the relationship between mindfulness exercise and self-control (H4), while self-control plays a mediating role in the relationship between mindfulness exercise and MH (H5). In addition, serial mediation analysis showed that mindfulness exercise indirectly affected MH through the path of sleep regularity and self-control (ME → SR → SC → MH), thus supporting H6. These results suggest that the association between mindfulness exercise and mental health may reflect a continuous regulatory process involving behavioral and psychological regulation, rather than a single isolated mechanism.

From the perspective of self-regulation, the sequence mediation pathway identified in this study can be interpreted as a hierarchical regulatory cascade. Mindfulness practice can be used as an upstream attentional regulation input to promote greater behavioral consistency (i.e., sleep regularity) in daily life (Xie et al., 2023). Stable behavioral regulation in turn supports physiological recovery and executive function, thereby strengthening psychological regulation in the form of self-control. Enhanced self-control is associated with more adaptive emotional function and better mental health outcomes. This multi-level explanation is closely related to the theory of self-regulation, which conceptualizes regulation as an interconnected system that spans the behavioral, physiological and psychological domains.

Previous studies have consistently shown that ME based interventions and physical and mental exercise are positively correlated with MH outcomes (Johnson et al., 2023; Niedermeier et al., 2022), including reducing college students’ psychological distress and enhancing well-being (Melnyk et al., 2020). However, most of the literature mainly emphasizes direct relationships or focuses on isolated mediating mechanisms, such as emotional regulation, perceived stress, or sleep quality (Alhawatmeh et al., 2022; Smith et al., 2024; Talley and Shelley-Tremblay, 2020). Although these studies provide valuable evidence on the effectiveness of mindfulness-related practices, they are often insufficient to explain how multiple self-regulation processes work together to affect mental health.

In contrast, this study adopts a more comprehensive perspective, proposes and empirically tests a sequential mediation model involving sleep regularity and self-control. This method expands the previous research and proves that the influence of ME on MH is carried out through a continuous adjustment process, rather than through a single psychological approach. By explicitly modeling the orderly relationship between behavioral regulation (sleep regularity) and psychological regulation (self-control), this study provides a more nuanced explanation for the link mechanism between mindfulness exercise and mental health.

In addition, the existing sleep-related research in the field of MH mainly focuses on sleep quality, sleep time or insomnia symptoms, and often regards sleep as a static or result-oriented variable (Palagini et al., 2022; Wang F. et al., 2024). In contrast, this study emphasizes that sleep regularity is a unique and theoretically meaningful construct that reflects an individual’s ability to maintain consistent daily activities. From the perspective of self-regulation, sleep regularity is a continuous behavior pattern formed by attention control, habit formation and daily self-discipline, rather than an instant evaluation of sleep experience.

The results of this study show that a stable sleep wake rhythm is beneficial not only in physiology, but also in psychology, especially in supporting self-regulation. This extends previous work by positioning sleep patterns as a positive behavioral pathway through which mindfulness practice can enhance self-control and help improve mental health outcomes. Compared with the research focusing solely on sleep quality, the results of this study emphasize the importance of considering the temporal stability and daily consistency of sleep behavior when investigating college students’ self-regulation and mental health.

In conclusion, this study complements and expands the existing literature by incorporating mindfulness exercises, sleep regularity and self-control into a unified sequence framework. It shifts the field from a fragmented interpretation to a more comprehensive understanding of how mindfulness-related movements exert mental health benefits through interconnected behavioral and psychological adjustment processes.

### 5.1 Theoretical implications

This study has made important theoretical contributions to the study of mindfulness training, self-regulation and mental health of college students.

This study has made important theoretical contributions to the literature research on mindfulness exercise, self-regulation and mental health of college students. First, these findings deepen the theoretical understanding of the relationship between mindfulness practice and mental health by clarifying the multi-level self-regulation mechanism. This study did not conceptualize mindfulness practice as a direct determinant of mental health outcomes (González-Martín et al., 2023), but pointed out that the association between mindfulness practice and mental health was achieved through coordinated behavior and psychological adjustment process. By identifying sleep regularity and self-control as sequential mediating variables, this study demonstrates how embodied attention practice is linked to downstream psychological functions through a hierarchical regulatory approach, thus advancing the theory of self-regulation.

Secondly, this study positioned the sleep pattern as a dynamic behavioral self-regulation structure, rather than just a sleep-related outcome variable, thus advancing the conceptualization of sleep patterns. Although previous studies mainly focused on sleep duration, sleep quality or insomnia symptoms (Palagini et al., 2022; Wang F. et al., 2024), sleep regularity in this framework represents a continuous behavior pattern, reflecting the stability of circadian rhythm, daytime consistency and daytime regulation. This study broadened the theoretical role of sleep in the theory of self-regulation and emphasized the fundamental role of sleep in maintaining high-order executive control by viewing sleep patterns as an observable form of behavioral self-regulation.

Third, the results emphasize the interdependence between daily behavior rhythm and high-order executive function (Sun et al., 2025). From the perspective of the regulatory system, stable behavioral procedures, such as a consistent sleep wake cycle, support physiological recovery and cognitive stability, thereby enhancing the persistence of the executive process, including impulse suppression and goal orientation. This explanation emphasizes that psychological self-control is not only a stable trait, but also a downstream regulatory ability, which is embedded in a broader pattern of behavioral consistency. This cross level regulatory integration enhances the systematicness of self-regulation theory and provides empirical support for its hierarchical structure.

Fourth, this study extends the self-regulation theory to non clinical university groups. Most of the existing literatures focus on clinical samples, developmental psychopathology or high-risk groups. The results show that self-regulation motivation plays a role not only in the pathological environment, but also in the conventional development environment. This expands the ecological applicability of self-regulation theory and supports its relevance to preventive mental health research.

Finally, by integrating mindfulness practice, sleep regularity and self-control into a unified sequence framework, this study transforms the literature from a fragmented mediation model to a system oriented regulatory perspective. The model conceptualizes behavioral and psychological regulation as a hierarchical component of the regulation cascade, rather than independently examining isolated mediators. This comprehensive approach provides a more coherent theoretical framework to understand how mindfulness related practices are statistically linked to mental health outcomes.

### 5.2 Practical implications

The research results have certain practical significance to promote the mental health of college students. First, the results of this study show that mindfulness practice may represent a promising and scalable approach for supporting students’ mental health (González-Martín et al., 2023; Zuo et al., 2023). Universities may consider incorporating mindfulness based exercise modules such as mindfulness exercise, breath centered physical activities, or awareness based exercise programs into physical education courses, health courses, or campus mental health programs. Compared with traditional psychological intervention, mindfulness training requires less professional resources and is more easily accepted by students in non clinical environment.

Secondly, this study emphasizes that sleep regularity is a MH intervention measure (Castiglione-Fontanellaz et al., 2023; Li et al., 2025). The campus health plan should not only emphasize the sleep time or sleep quality, but also promote a consistent sleep wake process. Practical strategies may include sleep hygiene education, focusing on regular bedtime and wake-up time, using digital tools or mobile apps to monitor sleep schedules, and incorporating sleep routine guidance into mindfulness exercise programs.

Third, the results emphasize the importance of SC as a kind of adjustable psychological resource. The intervention measures combining mindfulness training with structured daily behavior can indirectly improve students’ self-control ability, so as to improve emotion regulation and stress management (Liu et al., 2022; Zhang and Zhang, 2023). College counselors and educators can design courses that explicitly link mindfulness practice, sleep habits and goal-directed behavior to help students develop sustainable self-regulation.

Finally, the sequential mechanism determined in this study shows that comprehensive and multi group intervention may be more effective than single intervention. At the same time, mental health promotion based on mindfulness training, sleep mode and self-control development may have more stable and lasting benefits. The comprehensive framework provides a practical blueprint for colleges and universities to seek preventive mental health support and promote students’ long-term mental health.

### 5.3 Limitations and future research directions

It should be admitted that there are some limitations in this study. First, cross-sectional design limits causal inferences about the sequential relationship between mindfulness exercise, sleep regularity, and SC and MH. Although the proposed model has a good theoretical basis, future research should use longitudinal, experimental, or diary-based designs to verify the time series and causal dynamics of these pathways.

Secondly, all structures were assessed using self-reported measurements. Although there are procedural and statistical controls, this may increase the risk of common method bias. Future research can benefit from a variety of methods, such as combining objective sleep indicators (such as imaging devices or wearable devices, etc.), self-control behavior tasks, or multi-information mental health assessments to improve the robustness of measurements.

Thirdly, it should also be acknowledged that the “exercise mindfulness scale” was originally developed in a sports environment. Although the psychometric characteristics of the current university sample are satisfactory, future research can consider developing or adjusting a specially validated exercise mindfulness scale for the general population to further enhance structural specificity.

Finally, the sample is limited to college students with a single cultural background, which may limit the generalization of the research results. It is necessary to carry out in different cultures, ages and educational environments. In addition, future research can expand the existing framework by studying other mediators or regulatory variables (such as stress exposure, emotion regulation strategies or academic requirements), and strengthen the impact of practice by testing mindfulness exercise, sleep regularity and self-control.

## 6 Conclusion

Mindfulness exercise was found to be positively associated with mental health, and this association operated through sleep regularity and self-control. The results enhance the theoretical understanding of the behavioral and self-regulation mechanisms linking mindfulness exercise and mental health, and provide practical implications for the development of integrated mental health promotion interventions targeting sleep behavior and self-regulation in higher education environments.

## Data Availability

The original contributions presented in the study are included in the article/supplementary material, further inquiries can be directed to the corresponding author.
